# Prevalence and Socioeconomic Correlates of Adult Obesity in Europe: The Feel4Diabetes Study

**DOI:** 10.3390/ijerph191912572

**Published:** 2022-10-01

**Authors:** Dimitrios V. Diamantis, Kalliopi Karatzi, Paris Kantaras, Stavros Liatis, Violeta Iotova, Yulia Bazdraska, Tsvetalina Tankova, Greet Cardon, Katja Wikström, Imre Rurik, Emese Antal, Alelí M. Ayala-Marín, Natalia Giménez Legarre, Konstantinos Makrilakis, Yannis Manios

**Affiliations:** 1 Department of Nutrition and Dietetics, School of Health Science and Education, Harokopio University, 17671 Athens, Greece; 2 Laboratory of Dietetics and Quality of Life, Department of Food Science & Human Nutrition, School of Food and Nutritional Sciences, Agricultural University of Athens, 11855 Athens, Greece; 3 Medical School, National and Kapodistrian University of Athens, 11527 Athens, Greece; 4 Department of Paediatrics, Medical University of Varna, 9010 Varna, Bulgaria; 5 Clinical Center of Endocrinology and Gerontology, Medical University of Sofia, 1431 Sofia, Bulgaria; 6 Department of Movement and Sports Sciences, Ghent University, 9000 Ghent, Belgium; 7 Department of Public Health and Welfare, Finnish Institute for Health and Welfare, 00271 Helsinki, Finland; 8 Institute of Public Health and Clinical Nutrition, University of Eastern Finland, 70210 Kuopio, Finland; 9 Department of Family Medicine Hungarian Society of Nutrition, Semmelweis University, 1085 Budapest, Hungary; 10 Hungarian Society of Nutrition, 1088 Budapest, Hungary; 11 Growth, Exercise, Nutrition and Development (GENUD) Research Group, Facultad de Ciencias de la Salud, Universidad de Zaragoza, 50009 Zaragoza, Spain; 12 Instituto de Investigación Sanitaria Aragón (IIS Aragón), 50013 Zaragoza, Spain; 13 Instituto Agroalimentario de Aragón (IA2), 50009 Zaragoza, Spain; 14 Institute of Agri-Food and Life Sciences, Hellenic Mediterranean University Research Centre, 71410 Heraklion, Greece

**Keywords:** obesity, education, income security, occupation, socioeconomic factors

## Abstract

To effectively tackle obesity, it is necessary to identify all specific socioeconomic factors which contribute to its development. We aimed to highlight the prevalence of adult overweight/obesity in European countries and investigate the association of various socioeconomic factors and their accumulative effect on overweight/obesity status. Cross-sectional data from the Feel4Diabetes study for 24,562 adults residing in low socioeconomic areas were collected, representing Belgium, Finland, Greece, Spain, Bulgaria, and Hungary. Socioeconomic Burden Score (SEBS) was created, accounting for unemployment, financial insecurity, and education ≤ 12 years. Data were analyzed using analysis of variance and logistic regression. In total, 19,063 adults with complete data were included (34.5% overweight and 15.8% obese). The highest overweight/obesity rates occurred in Greece (37.5%/17.8%) and Hungary (35.4%/19.7%). After adjusting for confounders, age of <45 years and female sex were inversely associated with overweight/obesity, while low educational level (≤12 years), unemployment, and financial insecurity were positively associated. The increase in SEBS (clustering of socioeconomic disadvantages) was associated with increased overweight/obesity likelihood. This association of SEBS scores with overweight/obesity was evident for males and females across all examined countries, excluding males in low-income countries (Bulgaria and Hungary), where the highest SEBS score was inversely associated with overweight/obesity. The clustering burden of socioeconomic disadvantages on overweight/obesity was found to be influenced by the countries’ economic state and sex.

## 1. Introduction

Overweight and obesity are responsible for approximately USD 1 trillion worldwide of annual healthcare costs [1]. In Europe, this corresponds to about USD 220 billion and about 13.6 % of total healthcare expenditures [1]. This is of no surprise, as high BMI is evident in more than half of European adults and is associated with several comorbidities that affect all human organ systems [2,3]. The impact of obesity is not just physiological, but also negatively affects productivity, anxiety, depression, and mental well-being in general, which is significantly hindered by stigma and discrimination [4,5]. Obesity can be prevented through a healthy lifestyle promoted by effective public health policies [3]. However, pinpointing the population groups with increased risk of obesity is crucial in order to design the most effective prevention programs [6].

Socioeconomic status can be defined as a combined measure of an individual’s social and economic status [7]. Individuals or households of low socioeconomic areas are at higher risk of suffering from negative health consequences and of having inadequate resources to cope with the aforementioned inequality [7,8]. Consequently, their lives tend to be more negatively impacted by unexpected adverse health events or unexpected healthcare costs [9]. Obesity is closely linked with low socioeconomic areas in Europe, arising a concern regarding health inequalities in all countries’ policymakers [10,11]. Among the associated socioeconomic risks of obesity, many are more prevalent in low socioeconomic areas, such as limited educational attainment [12]. In contrast, financial constraints/low income and unemployment, which are more prevalent in these areas, tend to have a more complicated connection with obesity [13,14]. Accounting for all the obesity correlates, and their accumulative effect is crucial in understanding which population group is at higher risk of obesity. 

Understanding the obesity prevalence in low socioeconomic areas of Europe and the characteristics of the population groups that are in greater danger for this health problem is necessary for designing effective interventions to tackle the inequalities of obesity [6]. Thus, this study aims to present the overweight and obesity prevalence in low socioeconomic areas of six European countries and various associated socioeconomic and demographic factors of obesity. Furthermore, it was aimed to evaluate the accumulative effect of various socioeconomic factors on overweight/obesity, between different regions and different sexes.

## 2. Materials and Methods

### 2.1. Study Design

Baseline data collected from the European-Union-funded study “Feel4Diabetes” were utilized in the present study. The Feel4Diabetes study developed a school- and community-based intervention aiming at preventing Type 2 Diabetes among vulnerable groups of families in Europe and evaluated the intervention after implementation. A detailed description of the study can be found elsewhere [15,16]. The development of the study’s design took place in 2015, recruitment and baseline measurements took place in January to June 2016, intervention occurred between September 2016 and March 2017 and follow-up continued up until the June of 2018 [16]. The study was registered at clinicaltrials.gov as NCT02393872.

### 2.2. Setting and Participants

The data collection took place in six European countries and lasted three months (April–June 2016) [16]. The participating countries were categorized in three categories, based on the World Bank country classification derived from the 2013 Gross National Income per capita [17]. Bulgaria and Hungary were classified as low- to middle-income countries (LMIC), Finland and Belgium as high-income countries (HIC), while Greece and Spain were classified as HIC under strict economic/austerity measures. To identify “vulnerable” areas for the development of type 2 diabetes, low socioeconomic regions in HICs were selected, as these areas are associated with increased risk of diabetes development. 

To pinpoint the low socioeconomic areas, in each selected province, the school districts, municipalities, or other equivalent units were categorized in tertiles based on socioeconomic status indices (i.e., unemployment rates or literacy) acquired from data from official resources or authorities [18,19,20,21]. From the tertile with the lowest socioeconomic status indices, vulnerable areas were randomly selected. On the other hand, any school district, municipality, or other equivalent unit on the LMICs was considered as a vulnerable area, since the prevalence of type 2 diabetes is higher in LMICs [22].

### 2.3. Study Sample

The sample, initially, consisted of 24,562 parents/carers. Due to the small representation of the “underweight” population, these individuals were excluded from the current study (*n* = 603, 2.5%). All the participants with incomplete data on at least one confounder (sex, age group, education, occupation, and income insecurity status) and weight, height, and country of stay were excluded (*n* = 4896). In total 19,063 participants with complete data on all the aforementioned variables of interest were included in the analyses.

### 2.4. Measurements

The entry points to the “vulnerable” areas were the randomly selected schools and during the baseline measurements, a self-completed questionnaire was provided to be completed at home by the parents/carers of the children. Parents were asked to complete data around their age group, self-reported weight and height, sex, occupational status, education level, and income insecurity. The BMI was calculated for each individual using the Quetelet’s equation (weight (kg)/height (cm)^2^). The International Obesity Task Force (IOTF) cutoff points were utilized to categorize the participants as underweight (BMI < 18.5), normal weight (18.5–24.9), overweight (25.0–29.9) or obese (≥30). The region of residence (HIC, LMIC, or HIC under austerity measures) was not self-reported.

The occupational status was divided into two categories: employed, that included individuals working full/part-time, being retired or being full-time students; and unemployed individuals, that included unemployed or stay-at-home parents. The educational level was assessed by the reported duration of individuals’ studies and categorized to ≤12 and >12 years of education. The income insecurity was assessed by asking the following question: “Considering the total income in this household, how difficult or easy is it to cover your costs?”. The income insecurity status was coded based on the answers provided into two categories: easy/ease in covering costs (very easy, easy, or fairly easy) and difficult/difficulty in covering costs (fairly difficult, difficult, or very difficult).

### 2.5. The Socioeconomic Burden Score (SEBS)

The occupational status, educational attainment, and income insecurity are known predictors for the development of obesity. For the needs of the statistical analysis, a SEBS score was created for each participant by accounting for all three factors. More specifically, the SEBS score was calculated by adding 1 point each time a participant indicated one of the following socioeconomic disadvantages: education ≤ 12 years, unemployment and/or income insecurity (answer: difficult), with a minimum score of 0 and a maximum of 3. 

### 2.6. Ethical Approval

All the participating countries received ethical approval from the corresponding ethical committees; i.e., Belgium: Ethical committee of Ghent University (ethical approval code: B670201524437); Finland: Ethics Committee of THL (174/1801/2015); Greece: Ethics Committee of Harokopio University of Athens, the Greek Ministry of Education, Research and Religious Affairs and the Municipalities of Kallithea, Peristeri, Piraeus and Keratsini-Drapetsona (46/3-4-2015); Hungary: Bioethics Committee of University of Debrecen (20095/2016/EKU); Bulgaria: Medical University of Varna and the Municipality of Sofia (52/10-3-2016); and Spain: CEICA (Comité Ético de Investigación Clíinica de Aragóonde) (CP03/2016). All the participants received adequate information about the study’s design and aims, in the form of an information letter, and then signed a written informed consent. This study followed every applicable institutional regulation regarding the ethical use of human volunteers.

### 2.7. Statistical Analysis

All statistical analyses were performed using the SPSS software version 25.0 (Statistical Package for Social Sciences, SPSS Inc., Chicago, IL, USA). All categories are presented as number of participants along with the corresponding percentage. Descriptive statistics were computed for each country and, in total, sample and significant differences between the countries were computed using Pearson’s chi-square test. Descriptive statistics for years of education, employment, income insecurity and SEBS score were further subdivided according to weight status.

The association between various sociodemographic factors and the likelihood of being overweight or obese, compared to normal weight were examined in binary logistic regression models. An adjusted logistic regression model, based on the prior univariate analyses, was created by including all the aforementioned factors as confounders following forward model selection (i.e., age group, sex, educational attainment, occupational status, income insecurity, and region). A bivariate correlation matrix of the variables used can be found in the Appendix A (Table A1).

Another two logistic regression models (one univariate and one multivariate, independently for sex and age group) were executed to evaluate the association between SEBS score and overweight or obese status for each economic state (LMIC, HIC, HIC under austerity measures) and in the total sample. Finally, after accounting for the interaction between sex and SEBS score in the last two models (Table A2), two new models were created stratified by sex, with the latter being adjusted for age group.

## 3. Results

The study sample comprised 19,063 parents/carers from families in vulnerable areas. The descriptive characteristics of each country and the total sample are presented in Table 1. The total overweight and obesity rates were 34.5% and 15.8%, respectively (after including the underweight individuals, the corresponding rates were 33.7% and 15.4%). The majority of participants in each country were below the age of 45 (ranging from 78.0% to 89.6% between countries and 84.6% in total). The educational status of participants varied between countries. In Spain and Finland, only 4.9% and 10.1% of the participants had less or equal to 12 years of education, while Greece and Hungary had their participants almost equally distributed between ≤12 and >12 years of education (≤12 years: 49.7% and 47.4%, respectively). Unemployment rates were higher in Hungary and Greece (35.6% and 26.4%, respectively), along with income insecurity (75.6% and 72.7%, respectively). Both unemployment rate and income insecurity were evidently lower in HICs. Overweight incidence rates ranged from 32.4% (Belgium) to 37.5% (Greece). Obesity rates were higher among LMICs and Greece, with Hungary presenting the highest rates (19.7%).

The prevalence of overweight and obesity among all countries was higher in individuals with less education (less than or equal to 12 years) (overweight 38.0% and obesity 18.9%), than those with more than 12 years (overweight 32.9%, and obesity 14.2%) (Table 2). Obesity prevalence was lower in employed individuals (15.3%) compared to unemployed (17.5%), but overweight was higher in employed individuals (35.0% and 32.8%, respectively). Among participants with income insecurity, both overweight and obesity prevalence were higher (35.5% and 18.9%) than among those not reporting income insecurity (33.7% and 12.9%). Overall, the combined overweight and obesity prevalence was higher as SEBS score increased (score 0: 44.1%), (score 1: 51.9%), (score 2: 57.2%), but slightly decreased at a score equal to 3 (52.6%).

In the adjusted models (Table 3), age <45 years (OR 0.71; 95% CI 0.65, 0.78) and female sex (OR 0.24; 95% CI 0.22, 0.25) were found to be inversely associated with the likelihood of being overweight or obese. Having less than or equal to 12 years of education (OR 1.21; 95% CI 1.13, 1.29), being unemployed (OR 1.29; 95%CI 1.19, 1.39) and experiencing income insecurity (OR 1.37; 95%CI 1.28, 1.47) increased the overweight/obesity likelihood in the adjusted models. The association between the economic status of a country and the overweight/obesity status did not remain significant, after adjusting for all the aforementioned factors (*p* = 0.670).

A SEBS score of 1 or 2 or 3 compared to 0 was positively associated with an elevated likelihood of being overweight or obese in all the countries, even after adjusting for age group and sex (Table 4). This likelihood for SEBS scores of 1, 2, or 3 did not differ between the countries, based on the overlapping 95% CI. On the total sample, after adjusting for age group and sex, the likelihood of being overweight or obese was higher as the SEBS score increased, with individuals with score 1, 2 and 3 having OR 1.43 (95% CI 1.33, 1.54), 1.76 (1.62, 1.92) and 1, 99 (1, 76, 2,24).

After stratifying for sex in the total sample (Table 5), as the SEBS score was higher, so did the likelihood of overweight/obesity among women (SEBS score 1: OR 1.44 (95% CI 1.30, 1.59), 2: 1.96 (1.75, 2.20), 3: 2.27 (1.97, 2.62)); while for men, there was no similar increase (SEBS score 1: OR 1.44 (95% CI 1.30, 1.60), 2: 1.56 (1.38, 1.76), 3: 1.43 (1.14, 1.79)). A similar increase in SEBS score was associated with a higher obesity or overweight likelihood among women in all economic regions. Among high-income regions (with and without austerity measures), the likelihood of being overweight or obese indicated a potential increase as SEBS score elevated among men. In low-income countries, a reverse association with SEBS score and increased weight was evident only among those with the highest burden (SEBS score 3, OR: 0.70; 95% CI 0.52, 0,95).

## 4. Discussion

In total, about half of the participating adults from low socioeconomic areas of six European countries were overweight or obese, while about one in six to seven adults were obese. In the total sample, about one in three was educated for at most 12 years, one in five was unemployed or a stay-at-home parent, and one in two individuals was economically insecure. The rates of increased BMI, educational level, employment, and economic insecurity varied among countries. Age above 45 years, male sex, education ≤ 12 years, unemployment, and income insecurity were positively associated with increased BMI. The region of residence (countries’ economic status) was not associated with increased BMI, after adjusting for confounders. A high SEBS score (being unemployed, having ≤ 12 years of education and/or being income insecurity), predicted an accumulated elevated likelihood of overweight or obesity status, across all countries, compared to low SEBS. This effect was more evident among women in all countries. However, for men, this association varied from positive in high-income countries to negative in low-income countries. Overall, socioeconomic status remains a significant predictor of increased BMI in low socioeconomic areas of Europe. An accumulative effect is evident as the more the negative socioeconomic characteristics an individual has, the higher the odds of increased BMI, excluding men across low-income developed countries. The SEBS score can be utilized to account for this accumulative effect across different European countries.

Across the European Union members, overweight and obesity rates were estimated to be about 36.8% and 15.2%, respectively, in 2017 [23]. Our study, conducted in 2016 among low socioeconomic areas of Europe, provided a similar overall rate of BMI above 25, but a higher rate of obesity and a lower rate of overweight status. This finding can be attributed to the elevated rate of obesity across low socioeconomic areas [10]. From a further comparison with the 2014 and 2019 data from 18 to 64 years of age of Eurostat, we found out that among high-income countries (Belgium and Finland) and Spain, the obesity rates that our study provided were lower and among Greece higher than expected. The obesity rates of Bulgaria and Hungary, where the socioeconomic level was not accounted for during sampling, were similar to the expected rates [23]. 

The aforementioned role of the increased age group, male sex, and lower educational level on the increased likelihood of overweight/obesity in developed countries has been confirmed by various studies [10,12]. The mentioned connection was evaluated in a prior study, following a similar sampling methodology to ours, which suggested an increased likelihood of obesity among males, parents of high age, and low educational attainment only among females [24]. In contrast to our study, where educational attainment’s link with obesity was independent of sex, the aforementioned study compared completely illiterate individuals with literate and did not account for modifiers of this association, such as both sex and country’s economic development, that are known modifiers [12]. On the contrary, the direction of effect of unemployment and income with obesity is debated in the literature [13,14,25,26,27,28]. A longitudinal Finnish study has indicated that early long-term unemployment was associated with obesity at 31 years of age only among females, while obesity did not predict future unemployment in any sex [25]. Job loss and job-seeking, only among non-smokers, were also linked with increased weight status [14,28]. 

The relation of income and obesity is complex and suggests a reverse causality in the literature, especially among women [13], a phenomenon that can be attributed to the obesity stigma in job seeking, that is more prevalent among women [29,30]. Contradictory to income, economic/income insecurity has been linked with increased weight status [31,32,33]. However, economic insecurity was defined as the probability of experiencing a severe negative economic shock, often caused by job loss, or as unemployment. A prospective study, conducted among Australian adults, has shown that financial stress is associated with obesity, independently of income [34]. The financial stress was measured through a set of questions, evaluating the inability to pay for necessary expense, ask for financial aid and having shortage of money, in contrast with our study which asked a more direct question to evaluate economic insecurity. The novelty of this study is that it has provided evidence of the link between the perceived economic insecurity and overweight/obesity. Literature has indicated that stress, that can potentially be attributed to financial insecurity, is associated with obesity, through both physiological mechanisms and behavioral patterns, such as the overconsumption of highly palatable foods high in fat or sugar [35,36,37].

The socioeconomic status indicators used for the socioeconomic score calculation varied among the studies on obesity prevalence [38,39,40]. A review that utilized data from 333 studies, has indicated that there is a clear inverse association of socioeconomic status indicators in high-income countries with obesity, especially among women [38], meaning that the more vulnerable and socially deprived individuals have higher odds of being obese, confirming our findings. However, a more recent metanalysis has indicated that in developed countries, life-course socioeconomic score is associated with obesity only among women and not men [39]. Obesity was also associated with low socioeconomic level in both sexes, but overweight was associated significantly only for men, in South Wales [40]. Our findings only partly support those produced by the Newton et al. [39] study about the accumulated role of socioeconomic status across females, but opposing findings for men. Overall, men with socioeconomic deprivation had increased risk for overweight/obesity, but after accounting for the economic status of their country, this positive association was seen only in high-income and high-income-under-austerity-measures countries, while in low- to middle-income countries, the association was not seen or was even negative for the most socioeconomically deprived men. Accounting for this difference can shed light on the variation in the accumulated role of socioeconomic status on obesity in men. While the effect on women is more evident, there is a debate about the accumulative effect of socioeconomic background in men. In men, studies across developing countries of low income have indicated a positive association with high socioeconomic status (less socioeconomic burden) and obesity, while this connection is mixed in middle-income countries [41]. Meanwhile, a recent meta-analysis on developed countries has reported no significant association of life course socioeconomic status and obesity in men [39]. However, this meta-analysis has not accounted for this effect on the most burdened men and on developed countries of lower income. Such disparities in the effect of socioeconomic deprivation and the inverse association with obesity in men of high socioeconomic deprivation can be partially explained by the frequent risky single-occasion drinking and high risk of alcohol-use disorders of socioeconomically deprived men [42,43]. Moreover, such findings are indicative of limited food availability and limited food overconsumption across socioeconomically deprived men, with no personal financial source.

Low socioeconomic status is closely linked with increased weight status [38], and according to our results, the more the negative predictors an individual has, the higher the risk of increased weight. In such areas, individuals are more likely to have limited educational attainment and be economically insecure, and consequently be more heavily affected by health inequalities [7,8] and be unable to cope with financial constraints [13,14]. Such income inequalities and insecurities can promote overeating and weight gain through elevated stress [44,45]. After all, the effect of low socioeconomic status on obesity is mediated through emotional eating, uncontrolled eating, and psychological distress [46,47]. Another factor that is included in this equation is the limited financial availability for food, which can lead to limited consumption of nutritionally rich foods, and elevated availability for nutritionally poor and energetically high food options in low-socioeconomic groups [48]. Accounting for the increasing trend in overweight and obesity through Europe, lower socioeconomic areas are at greater risk of not only developing but also maintaining the high obesity prevalence [23]. Identifying those individuals of increased risk of obesity, while accounting for sex differences, can result in more effective and targeted approaches to tackle this phenomenon [6].

A strength of the current study is that by collecting data in low socioeconomic areas of developed countries, we were able to identify a sample with a high incidence of unemployment, low educational background, and economic insecurity. Thus, we were able to increase the sample distribution across the various socioeconomic levels, which is a common limitation of studies in the literature [39]. Another strength is that for the sake of the statistical analysis, we have created a score accounting for three simple parameters of socioeconomic burden, a score that was associated with the likelihood of increased weight status. 

This study has also a set of limitations. First and foremost, the observational nature of the current study has prevented us from establishing causal relationships. Additionally, due to selecting schools as entry points to the community, we have included only adult parents or carers of young children in Europe, and thus not a representative sample of the population. Furthermore, for high-income countries, the sample includes individuals residing only in low socioeconomic areas, hence limiting the generalization of our findings. However, the representativeness of working-age adults is considered high, due to the large sample size. Furthermore, by including individuals from low socioeconomic areas, we were able to have a high representation of unemployed, economically insecure, and inadequately educated adults, increasing the strength of our resulted associations. The reported associations were not adjusted for alcohol consumption, nutrition literacy, yearly income, and availability of healthy food options, as no pertinent data were available. Reporting bias might also be an issue due to self-reported questionnaires and especially for weight and height. However, evidence suggests BMI computed measures from self-reported weight and height among adults of various socioeconomic backgrounds is a valid measure [49]. Moreover, the SEBS score includes only three factors, namely education, financial insecurity, and employment. Although the literature suggests a profuse amount of obesity correlates, in this study, we only wanted to create a simple score to evaluate the risk of obesity by just using three significant correlates and utilizing sex and country of residence data. Finally, these variables were also checked for multicollinearity in a bivariate correlation matrix. Although many significant correlations can be found, no correlation indicated a Pearson coefficient higher than 0.30 or lower than −0.30 and, therefore, there was evidence of only negligible correlations [50]. 

## 5. Conclusions

Populations living in regions with low socioeconomic indexes across developed European countries have high rates of overweight/obesity. The existing negative sociodemographic factors (unemployment, income insecurity, and education) appear to have an accumulative effect in the prevalence of overweight/obesity that differs between sexes. Understanding the extent of those inequalities and creating targeted and tailor-made interventions for populations living in low socioeconomic regions might be a top priority issue for limiting social and health inequalities and effectively tackling the obesity epidemic, which is more prevalent among those populations.

## Figures and Tables

**Table 1:** Descriptive table of participants’ baseline characteristics.

Characteristics	Categories	Belgium	Finland	Greece	Hungary	Bulgaria	Spain	Total	*p*-Value
Participants (% of total sample)	-	3048 (16.0%)	2047 (10.7%)	3806 (20.0%)	3078 (16.1%)	4904 (25.7%)	2180 (11.4%)	19,063	<0.001
Age group	<45	2732 (89.6%)	1755 (85.7%)	2968 (78.0%)	2736 (88.9%)	4227 (86.2%)	1711 (78.5%)	16,129 (84.6%)	<0.001
	≥45	316 (10.4%)	292 (14.3%)	838 (22.0%)	342 (11.1%)	677 (13.8%)	469 (21.5%)	2934 (15.4%)	
Sex	Female	1605 (52.7%)	1147 (56.0%)	2054 (54.0%)	1668 (54.2%)	2518 (51.3%)	1169 (53.6%)	10,161 (53.3%)	<0.001
	Male	1443 (47.3%)	900 (44.0%)	1752 (46.0%)	1410 (45.8%)	2386 (48.7%)	1011 (46.4%)	8902 (46.7%)	
Education	≤12 years	781 (25.6%)	206 (10.1%)	1891 (49.7%)	1458 (47.4%)	1701 (34.7%)	107 (4.9%)	6144 (32.2%)	<0.001
	>12 years	2267 (74.4%)	1841 (89.9%)	1915 (50.3%)	1620 (52.6%)	3203 (65.3%)	2073 (95.1%)	12,919 (67.8%)	
Occupational status	Unemployed	377 (12.4%)	276 (13.5%)	1003 (26.4%)	1095 (35.6%)	853 (17.4%)	375 (17.2%)	3979 (20.9%)	<0.001
	Employed	2671 (87.6%)	1771 (86.5%)	2803 (73.6%)	1983 (64.4%)	4051 (82.6%)	1805 (82.8%)	15,084 (79.1%)	
Income insecurity	Difficulty in covering costs	515 (16.9%)	504 (24.6%)	2768 (72.7%)	2327 (75.6%)	2040 (41.6%)	915 (42.0%)	9069 (47.6%)	<0.001
	Ease in covering costs	2533 (83.1%)	1543 (75.4%)	1038 (27.3%)	751 (24.4%)	2864 (58.4%)	1265 (58.0%)	9994 (52.4%)	

Age group, sex, education, occupational status, and income insecurity display the number of participants and, in brackets, the incidence rate in the total population of the individual country. *p*-values were derived from analyses of variance.

**Table 2:** Descriptive table of participants’ baseline weight status based on sociodemographic characteristics.

Characteristics	Categories	Belgium	Finland	Greece	Hungary	Bulgaria	Spain	Total	*p*-Value
BMI	Overweight	986 (32.4%)	737 (36.0%)	1429 (37.5%)	1088 (35.4%)	1621 (33.1%)	720 (33.0%)	6581 (34.5%)	<0.001
	Obese	330 (10.8%)	334 (16.3%)	677 (17.8%)	607 (19.7%)	811 (16.5%)	243 (11.2%)	3002 (15.8%)	
Education of ≤12 years	Overweight	311 (39.8%)	82 (39.8%)	743 (39.3%)	493 (33.8%)	663 (39.0%)	41 (38.3%)	2333 (38.0%)	<0.001
	Obese	105 (13.4%)	44 (21.4%)	366 (19.4%)	271 (18.6%)	357 (21.0%)	21 (19.6%)	1164 (18.9%)	
Education of >12 years	Overweight	675 (29.8%)	655 (35.6%)	686 (35.8%)	595 (36.7%)	958 (29.9%)	679 (32.8%)	4248 (32.9%)	
	Obese	225 (9.9%)	290 (15.8%)	311 (16.2%)	336 (20.7%)	454 (14.2%)	222 (10.7%)	1838 (14.2%)	
Unemployment	Overweight	120 (31.8%)	93 (33.7%)	331 (33.0%)	390 (35.6%)	249 (29.2%)	122 (32.5%)	1305 (32.8%)	<0.001
	Obese	70 (18.6%)	56 (20.3%)	166 (16.6%)	213 (19.5%)	129 (15.1%)	63 (16.8%)	697 (17.5%)	
Employment	Overweight	866 (32.4%)	644 (36.4%)	1098 (39.2%)	698 (35.2%)	1372 (33.9%)	598 (33.1%)	5276 (35.0%)	
	Obese	260 (9.7%)	278 (15.7%)	511 (18.2%)	394 (19.9%)	682 (16.8%)	180 (10.0%)	2305 (15.3%)	
Income insecurity (difficulty in covering costs)	Overweight	198 (38.4%)	189 (37.5%)	1034 (37.4%)	823 (35.4%)	670 (32.8%)	302 (33.0%)	3216 (35.5%)	<0.001
	Obese	85 (16.5%)	108 (21.4%)	528 (19.1%)	473 (20.3%)	383 (18.8%)	140 (15.3%)	1717 (18.9%)	
Income security (ease in covering costs)	Overweight	788 (31.1%)	548 (35.5%)	395 (38.1%)	265 (35.3%)	951 (33.2%)	418 (33.0%)	3365 (33.7%)	
	Obese	245 (9.7%)	226 (14.6%)	149 (14.4%)	134 (17.8%)	428 (14.9%)	103 (8.1%)	1285 (12.9%)	
SEBS score (overweight and obese/sum of residents with the same score)	0 (no negative factors)	684/1825 (37.5%)	628/1273 (49.3%)	293/577 (50.8%)	199/400 (49.8%)	794/1762 (45.1%)	466/1112 (41.9%)	3064/6949 (44.1%)	<0.001
	1 (1 negative factor)	419/850 (49.3%)	330/587 (56.2%)	750/1344 (55.8%)	612/1062 (57.6%)	932/1877 (49.7%)	332/778 (42.7%)	3375/6498 (51.9%)	
	2 (2 negative factors)	169/296 (57.1%)	97/162 (59.9%)	771/1337 (57.7%)	601/1030 (58.3%)	599/1078 (55.6%)	138/251 (55.0%)	2375/4154 (57.2%)	
	3 (3 negative factors)	44/77 (57.1%)	16/25 (64.0%)	292/548 (53.3%)	283/586 (48.3%)	107/187 (57.2%)	27/39 (69.2%)	769/1462 (52.6%)	

BMI cells display the number of participants and in brackets the incidence rate in the total population of the individual country. Cells in Education per weight status, Occupational status per weight status, and Income insecurity per weight status include the number of overweight or obese participants in each country and in brackets the incidence rate in the population under the same category of the individual country. Cells in SEBS score represent the number of overweight and obese individuals/total cell sample of participants with the same SEBS score in the country (incidence rate in the country’s population with the same SEBS score). The SEBS score was calculated by adding 1 point each time a participant indicated one of the following negative factors: education ≤ 12 years, unemployment, income insecurity (answer: difficult), with a minimum of 0 and a maximum score of 3. *p*-values were derived from analyses of variance.

**Table 3:** Univariate and multivariate associations between sociodemographic risk factors and Overweight/Obesity risk.

Sociodemographic Risk Factors	Comparator	Reference	*Univariate/Unadjusted Models* OR (95% CI)	*p*-Value	*Multivariate/Adjusted Model* OR (95% CI)	*p*-Value
Age	<45 years	≥45 years	0.54 (0.49, 0.58)	<0.001	0.71 (0.65, 0.78)	<0.001
Sex	Female	Male	0.24 (0.23, 0.26)	<0.001	0.24 (0.22, 0.25)	<0.001
Education	≤12	>12 years	1.48 (1.40, 1.58)	<0.001	1.21 (1.13, 1.29)	<0.001
Occupational status	Unemployed	Employed	1.00 (0.93, 1.07)	0.950	1.29 (1.19, 1.39)	<0.001
Income insecurity	Difficulty in covering costs	Ease in covering costs	1.37 (1.29, 1.45)	<0.001	1.37 (1.28, 1.47)	<0.001
Countries’ economic status	Low income	High income	1.21 (1.13, 1.30)	<0.001	1.02 (0.94, 1.10)	0.670
	Under austerity measures	High income	1.19 (1.11, 1.29)	<0.001	0.98 (0.90, 1.07)	0.684

Values are presented as Odds Ratio (95% Confidence Interval). In the unadjusted models, variables are independently examined for their association with overweight/obesity likelihood. All variables in the adjusted model are included in one model; thus, each variable is adjusted for the rest. *p*-values were derived from logistic regression.

**Table 4:** SEBS score and the risk of being overweight or obese based on the country’s economic status.

Economic Region	SEBS Score (0 = Reference)	OR Unadjusted (95% CI)	*p*-Value	OR Adjusted (95% CI)	*p*-Value
High income (Belgium and Finland)	1	1.48 (1.31, 1.68)	<0.001	1.52 (1.34, 1.73)	<0.001
	2	1.89 (1.55, 2.30)	<0.001	2.06 (1.68, 2.53)	<0.001
	3	1.94 (1.30, 2.90)	0.001	2.43 (1.61, 3.66)	0.001
Under austerity measures (Greece and Spain)	1	1.27 (1.12, 1.45)	<0.001	1.43 (1.24, 1.64)	<0.001
	2	1.64 (1.43, 1.88)	<0.001	1.85 (1.59, 2.15)	<0.001
	3	1.46 (1.21, 1.76)	<0.001	2.33 (1.9, 2.85)	<0.001
Low income (Bulgaria and Hungary)	1	1.30 (1.17, 1.46)	<0.001	1.32 (1.17, 1.49)	<0.001
	2	1.56 (1.38, 1.76)	<0.001	1.53 (1.34, 1.74)	<0.001
	3	1.20 (1.02, 1.41)	0.031	1.71 (1.43, 2.04)	<0.001
Total sample	1	1.37 (1.28, 1.47)	<0.001	1.43 (1.33, 1.54)	<0.001
	2	1.69 (1.57, 1.83)	<0.001	1.76 (1.62, 1.92)	<0.001
	3	1.41 (1.26, 1.58)	<0.001	1.99 (1.76, 2.24)	<0.001

OR: Odds ratios, 95% CI: 95% Confidence Interval. The OR (95% CI) column represents the odds of being overweight or obese (95% Confidence Interval) based on the SEBS score value. The SEBS score was calculated by adding 1 point each time a participant indicated one of the following negative factors: education ≤ 12 years, unemployment, or income insecurity (answer: difficult), with a minimum of 0 and a maximum score of 3. Each participant was assigned one score. *p*-values were derived from logistic regression. The second model is adjusted for sex and age group.

**Table 5:** SEBS score and the risk of being overweight or obese based on the country’s economic status, divided by sex.

Economic Region	Sex	SEBS Score (0 = Reference)	OR Unadjusted (95% CI)	*p*-Value	OR Adjusted (95% CI)	*p*-Value
High income (Belgium and Finland)	Female	1	1.57 (1.31, 1.87)	<0.001	1.56 (1.31, 1.87)	<0.001
		2	2.29 (1.77, 2.97)	<0.001	2.30 (1.77, 2.98)	<0.001
		3	2.62 (1.62, 4.23)	<0.001	2.67 (1.65, 4.31)	<0.001
	Male	1	1.50 (1.24, 1.81)	<0.001	1.49 (1.23, 1.80)	<0.001
		2	1.82 (1.31, 2.53)	<0.001	1.76 (1.27, 2.45)	0.001
		3	1.95 (0.89, 4.27)	0.094	1.96 (0.90, 4.29)	0.092
Under austerity measures (Greece and Spain)	Female	1	1.48 (1.21, 1.80)	<0.001	1.47 (1.20, 1.79)	<0.001
		2	2.18 (1.77, 2.69)	<0.001	2.19 (1.77, 2.70)	<0.001
		3	2.45 (1.92, 3.13)	<0.001	2.46 (1.93, 3.15)	<0.001
	Male	1	1.42 (1.17, 1.73)	<0.001	1.42 (1.17, 1.73)	<0.001
		2	1.53 (1.24, 1.89)	<0.001	1.53 (1.23, 1.89)	<0.001
		3	2.30 (1.51, 3.51)	<0.001	2.29 (1.50, 3.50)	<0.001
Low income (Bulgaria and Hungary)	Female	1	1.65 (1.38, 1.97)	<0.001	1.64 (1.38, 1.96)	<0.001
		2	2.21 (1.83, 2.67)	<0.001	2.21 (1.83, 2.67)	<0.001
		3	2.74 (2.20, 3.41)	<0.001	2.75 (2.21, 3.43)	<0.001
	Male	1	1.04 (0.86, 1.24)	0.700	1.05 (0.87, 1.26)	0.611
		2	1.02 (0.84, 1.24)	0.833	1.03 (0.85, 1.25)	0.747
		3	0.69 (0.51, 0.94)	0.017	0.70 (0.52, 0.95)	0.020
Total sample	Female	1	1.45 (1.31, 1.60)	<0.001	1.44 (1.30, 1.59)	<0.001
		2	1.97 (1.76, 2.21)	<0.001	1.96 (1.75, 2.20)	<0.001
		3	2.27 (1.97, 2.62)	<0.001	2.27 (1.97, 2.62)	<0.001
	Male	1	1.45 (1.30, 1.61)	<0.001	1.44 (1.30, 1.60)	<0.001
		2	1.57 (1.40, 1.77)	<0.001	1.56 (1.38, 1.76)	<0.001
		3	1.44 (1.15, 1.80)	0.001	1.43 (1.14, 1.79)	0.002

OR: Odds ratios, 95% CI: 95% Confidence Interval. The OR (95% CI) column represents the odds of being overweight or obese (95% Confidence Interval) based on the SEBS score value. The SEBS score was calculated by adding 1 point each time a participant indicated one of the following negative factors: education ≤ 12 years, unemployment, or income insecurity (answer: difficult), with a minimum of 0 and a maximum score of 3. Each participant was assigned one score. *p*-values were derived from logistic regression. The second model is adjusted for sex and age group.

## Data Availability

The data presented in this study are available on request from the corresponding author. The data are not publicly available.

## Appendix A

**Table A1:** Bivariate correlations between age group, sex, education, employment and income security.

Sociodemographic Risk Factors	Age	Sex	Education	Occupational Status	Income Insecurity
Age	-	0.172 [<0.001]	−0.002 [0.818]	0.014 [0.052]	−0.026 [<0.001]
Sex	0.172 [<0.001]	-	−0.078 [<0.001]	0.185 [<0.001]	0.028 [<0.001]
Education	−0.002 [0.818]	−0.078 [<0.001]	-	0.169 [<0.001]	0.264 [<0.001]
Occupational status	0.014 [0.052]	0.185 [<0.001]	0.169 [<0.001]	-	0.170 [<0.001]
Income insecurity	−0.026 [<0.001]	0.028 [<0.001]	0.264 [<0.001]	0.170 [<0.001]	-

Values in cells represent the Pearson correlation coefficient and the corresponding [*p*-value]. Comparator Reference values for the socioeconomic factors are: ≥45 years (age), male (sex), >12 years (education), unemployment (occupational status), and ease in covering costs (income insecurity).

**Table A2:** SEBS score and the risk of being overweight or obese based on the country’s economic status. (Interaction term of sex * SEBS score.)

Economic Region	SEBS Score (0 = Reference)	OR Unadjusted (95% CI)	*p*-Value	OR Adjusted (95% CI)	*p*-Value
High income (Belgium and Finland)	1	1.50 (1.24, 1.81)	<0.001	1.48 (1.22, 1.79)	<0.001
	2	1.82 (1.31, 2.53)	<0.001	1.73 (1.25, 2.41)	0.001
	3	1.95 (0.89, 4.27)	0.094	1.97 (0.90, 4.31)	0.090
	1 * sex (female)	1.05 (0.81, 1.36)	0.733	1.06 (0.81, 1.37)	0.673
	2 * sex (female)	1.26 (0.83, 1.91)	0.280	1.32 (0.87, 2.01)	0.193
	3 * sex (female)	1.34 (0.54, 3.36)	0.528	1.34 (0.54, 3.37)	0.527
Under austerity measures (Greece and Spain)	1	1.42 (1.17, 1.73)	<0.001	1.42 (1.17, 1.73)	<0.001
	2	1.53 (1.24, 1.89)	<0.001	1.53 (1.23, 1.89)	<0.001
	3	2.30 (1.51, 3.51)	<0.001	2.29 (1.50, 3.50)	<0.001
	1 * sex (female)	1.04 (0.78, 1.37)	0.808	1.03 (0.78, 1.36)	0.830
	2 * sex (female)	1.42 (1.06, 1.92)	0.020	1.43 (1.06, 1.93)	0.018
	3 * sex (female)	1.07 (0.65, 1.74)	0.798	1.07 (0.66, 1.75)	0.773
Low income (Bulgaria and Hungary)	1	1.04 (0.86, 1.24)	0.700	1.05 (0.88, 1.26)	0.585
	2	1.02 (0.84, 1.24)	0.833	1.04 (0.85, 1.26)	0.721
	3	0.69 (0.51, 0.94)	0.017	0.70 (0.52, 0.95)	0.021
	1 * sex (female)	1.59 (1.24, 2.05)	<0.001	1.56 (1.21, 2.01)	0.001
	2 * sex (female)	2.16 (1.66, 2.83)	<0.001	2.13 (1.63, 2.79)	<0.001
	3 * sex (female)	3.95 (2.72, 5.74)	<0.001	3.92 (2.70, 5.69)	<0.001
Total sample	1	1.45 (1.30, 1.61)	<0.001	1.44 (1.30, 1.60)	<0.001
	2	1.57 (1.40, 1.77)	<0.001	1.56 (1.38, 1.76)	<0.001
	3	1.44 (1.15, 1.80)	0.001	1.43 (1.14, 1.79)	0.002
	1 * sex (female)	1.00 (0.86, 1.16)	0.994	1.00 (0.86, 1.15)	0.958
	2 * sex (female)	1.25 (1.06, 1.48)	0.008	1.26 (1.07, 1.49)	0.006
	3 * sex (female)	1.58 (1.21, 2.06)	0.001	1.59 (1.22, 2.07)	0.001

OR: Odds ratios, 95% CI: 95% Confidence Interval. The OR (95% CI) column represents the odds of being overweight or obese (95% Confidence Interval) based on the SEBS score value. The SEBS score was calculated by adding 1 point each time a participant indicated one of the following negative factors: education ≤ 12 years, unemployment, or income insecurity (answer: difficult), with a minimum of 0 and a maximum score of 3. Each participant was assigned one score. *p*-values were derived from logistic regression. The first model is adjusted for age. The interaction term is presented as SEBS score * sex (female). The second model is adjusted for sex and age group.

## References

[1] Lobstein C.T., Brinsden H. (2020). Obesity: Missing the 2025 Global Targets. Trends, Costs and Country Reports.

[2] Eurostat Overweight and Obesity-BMI Statistics. https://ec.europa.eu/eurostat/statistics-explained/index.php?title=Overweight_and_obesity_-_BMI_statistics.

[3] World Health Organization Obesity and Overweight. https://www.who.int/news-room/fact-sheets/detail/obesity-and-overweight.

[4] Sarwer D.B., Polonsky H.M. (2016). The Psychosocial Burden of Obesity. Endocrinol. Metab. Clin. N. Am..

[5] Goettler A., Grosse A., Sonntag D. (2017). Productivity Loss Due to Overweight and Obesity: A Systematic Review of Indirect Costs. BMJ Open.

[6] Harvey J.R., Ogden D. (2014). Obesity Treatment in Disadvantaged Population Groups: Where Do We Stand and What Can We Do?. Prev. Med..

[7] Baker E.H. (2014). Socioeconomic Status, Definition. The Wiley Blackwell Encyclopedia of Health, Illness, Behavior, and Society.

[8] Smith J.P., Kington R. (1997). Demographic and Economic Correlates of Health in Old Age. Demography.

[9] Leonard T., Hughes A.E., Pruitt S.L. (2017). Understanding How Low-Socioeconomic Status Households Cope with Health Shocks: An Analysis of Multi-Sector Linked Data. Ann. Am. Acad. Pol. Soc. Sci..

[10] Loring B., Robertson A. (2014). Obesity and Inequities: Guidance for Addressing Inequities in Overweight and Obesity.

[11] Mohammed S.H., Habtewold T.D., Birhanu M.M., Sissay T.A., Tegegne B.S., Abuzerr S., Esmaillzadeh A. (2019). Neighbourhood Socioeconomic Status and Overweight/Obesity: A Systematic Review and Meta-Analysis of Epidemiological Studies. BMJ Open.

[12] Cohen A.K., Rai M., Rehkopf D.H., Abrams B. (2013). Educational Attainment and Obesity: A Systematic Review. Obes. Rev. Off. J. Int. Assoc. Study Obes..

[13] Kim T.J., Knesebeck O. (2018). von dem Income and Obesity: What Is the Direction of the Relationship? A Systematic Review and Meta-Analysis. BMJ Open.

[14] Hughes A., Kumari M. (2017). Unemployment, Underweight, and Obesity: Findings from Understanding Society (UKHLS). Prev. Med..

[15] Manios Y., Mavrogianni C., Lambrinou C.-P., Cardon G., Lindström J., Iotova V., Tankova T., Civeira F., Kivelä J., Jancsó Z. (2020). Two-Stage, School and Community-Based Population Screening Successfully Identifies Individuals and Families at High-Risk for Type 2 Diabetes: The Feel4Diabetes-Study. BMC Endocr. Disord..

[16] Manios Y., Androutsos O., Lambrinou C.-P., Cardon G., Lindstrom J., Annemans L., Mateo-Gallego R., de Sabata M.S., Iotova V., Kivela J. (2018). A School- and Community-Based Intervention to Promote Healthy Lifestyle and Prevent Type 2 Diabetes in Vulnerable Families across Europe: Design and Implementation of the Feel4Diabetes-Study. Public Health Nutr..

[17] World Bank World Development Indicators. https://openknowledge.worldbank.org/handle/10986/18237.

[18] ELSTAT Hellenic Statistical Authority (ELSTAT). https://www.statistics.gr/.

[19] Statistics Finland’s PxWeb Databases. https://pxnet2.stat.fi/PXWeb/pxweb/en/StatFin/StatFin__vrm__vaerak/?rxid=6b89654c-24b1-4509-816f-80b00b6e497f.

[20] Instituto Aragonés de Estadística (IAEST). https://www.aragon.es/inicio.

[21] VDAB-Arvastat. https://arvastat.vdab.be/.

[22] Agardh E., Allebeck P., Hallqvist J., Moradi T., Sidorchuk A. (2011). Type 2 Diabetes Incidence and Socio-Economic Position: A Systematic Review and Meta-Analysis. Int. J. Epidemiol..

[23] Eurostat Obesity Rate by Body Mass Index (BMI). https://ec.europa.eu/eurostat/databrowser/view/sdg_02_10/default/table?lang=en.

[24] Tzotzas T., Vlahavas G., Papadopoulou S.K., Kapantais E., Kaklamanou D., Hassapidou M. (2010). Marital Status and Educational Level Associated to Obesity in Greek Adults: Data from the National Epidemiological Survey. BMC Public Health.

[25] Laitinen J., Power C., Ek E., Sovio U., Järvelin M.R. (2002). Unemployment and Obesity among Young Adults in a Northern Finland 1966 Birth Cohort. Int. J. Obes. Relat. Metab. Disord. J. Int. Assoc. Study Obes..

[26] Morris S. (2007). The Impact of Obesity on Employment. Labour. Econ..

[27] Le Strat Y., Melchior M., Gorwood P., Tebeka S., Dubertret C. (2020). The Role of Comorbidity in the Association of Obesity with Unemployment and Disability. Ann. Epidemiol..

[28] Monsivais P., Martin A., Suhrcke M., Forouhi N.G., Wareham N.J. (2015). Job-Loss and Weight Gain in British Adults: Evidence from Two Longitudinal Studies. Soc. Sci. Med..

[29] Flint S.W., Čadek M., Codreanu S.C., Ivić V., Zomer C., Gomoiu A. (2016). Obesity Discrimination in the Recruitment Process: “You’re Not Hired!”. Front. Psychol..

[30] Mason K. (2012). The Unequal Weight of Discrimination: Gender, Body Size, and Income Inequality. Soc. Probl..

[31] Watson B. (2018). Does Economic Insecurity Cause Weight Gain among Canadian Labor Force Participants?. Rev. Income Wealth.

[32] Smith T.G., Stoddard C., Barnes M.G. (2009). Why the Poor Get Fat: Weight Gain and Economic Insecurity. Forum Health Econ. Policy.

[33] Barnes M.G., Smith T.G., Yoder J.K. (2013). Effects of Household Composition and Income Security on Body Weight in Working-Age Men. Obesity.

[34] Siahpush M., Huang T.T.-K., Sikora A., Tibbits M., Shaikh R.A., Singh G.K. (2014). Prolonged Financial Stress Predicts Subsequent Obesity: Results from a Prospective Study of an Australian National Sample. Obesity.

[35] Björntorp P. (2001). Do Stress Reactions Cause Abdominal Obesity and Comorbidities?. Obes. Rev. Off. J. Int. Assoc. Study Obes..

[36] la Fleur S.E., Houshyar H., Roy M., Dallman M.F. (2005). Choice of Lard, but Not Total Lard Calories, Damps Adrenocorticotropin Responses to Restraint. Endocrinology.

[37] Foster M.T., Warne J.P., Ginsberg A.B., Horneman H.F., Pecoraro N.C., Akana S.F., Dallman M.F. (2009). Palatable Foods, Stress, and Energy Stores Sculpt Corticotropin-Releasing Factor, Adrenocorticotropin, and Corticosterone Concentrations after Restraint. Endocrinology.

[38] McLaren L. (2007). Socioeconomic Status and Obesity. Epidemiol. Rev..

[39] Newton S., Braithwaite D., Akinyemiju T.F. (2017). Socio-Economic Status over the Life Course and Obesity: Systematic Review and Meta-Analysis. PLoS ONE.

[40] Ghosh A., Charlton K.E., Batterham M.J. (2016). Socioeconomic Disadvantage and Its Implications for Population Health Planning of Obesity and Overweight, Using Cross-Sectional Data from General Practices from a Regional Catchment in Australia. BMJ Open.

[41] Dinsa G.D., Goryakin Y., Fumagalli E., Suhrcke M. (2012). Obesity and Socioeconomic Status in Developing Countries: A Systematic Review. Obes. Rev. Off. J. Int. Assoc. Study Obes..

[42] Calling S., Ohlsson H., Sundquist J., Sundquist K., Kendler K.S. (2019). Socioeconomic Status and Alcohol Use Disorders across the Lifespan: A Co-Relative Control Study. PLoS ONE.

[43] Grittner U., Kuntsche S., Gmel G., Bloomfield K. (2013). Alcohol Consumption and Social Inequality at the Individual and Country Levels—Results from an International Study. Eur. J. Public Health.

[44] Offer A., Pechey R., Ulijaszek S. (2010). Obesity under Affluence Varies by Welfare Regimes: The Effect of Fast Food, Insecurity, and Inequality. Econ. Hum. Biol..

[45] Offer A., Pechey R., Ulijaszek S. (2009). Obesity: The Welfare Regime Hypothesis.

[46] Spinosa J., Christiansen P., Dickson J.M., Lorenzetti V., Hardman C.A. (2019). From Socioeconomic Disadvantage to Obesity: The Mediating Role of Psychological Distress and Emotional Eating. Obesity.

[47] Pigeyre M., Rousseaux J., Trouiller P., Dumont J., Goumidi L., Bonte D., Dumont M.-P., Chmielewski A., Duhamel A., Amouyel P. (2016). How Obesity Relates to Socio-Economic Status: Identification of Eating Behavior Mediators. Int. J. Obes..

[48] Pechey R., Monsivais P. (2016). Socioeconomic Inequalities in the Healthiness of Food Choices: Exploring the Contributions of Food Expenditures. Prev. Med..

[49] Hodge J.M., Shah R., McCullough M.L., Gapstur S.M., Patel A.V. (2020). Validation of Self-Reported Height and Weight in a Large, Nationwide Cohort of U.S. Adults. PLoS ONE.

[50] Hinkle D.E., Wiersma W., Jurs S.G. (2003). Applied Statistics for the Behavioral Sciences.

